# Extreme sensitivity in Snowball Earth formation to mountains on PaleoProterozoic supercontinents

**DOI:** 10.1038/s41598-019-38839-6

**Published:** 2019-02-20

**Authors:** Amber Walsh, Thomas Ball, David M. Schultz

**Affiliations:** 1 School of Physics and Astronomy, University of Manchester, Manchester, UK; 2 Present Address: Department of Meteorology, University of Reading, Reading, UK; 3 Present Address: Tyndall Centre for Climate Change Research, School of Environmental Sciences, University of East Anglia, Norwich, UK; 4 Centre for Atmospheric Science, School of Earth and Environmental Sciences, University of Manchester, Manchester, UK

## Abstract

During the PaleoProterozoic 2.45 to 2.2 billion years ago, several glaciations may have produced Snowball Earths. These glacial cycles occurred during large environmental change when atmospheric oxygen was increasing, a supercontinent was assembled from numerous landmasses, and collisions between these landmasses formed mountain ranges. Despite uncertainties in the composition of the atmosphere and reconstruction of the landmasses, paleoclimate model simulations can test the sensitivity of the climate to producing a Snowball Earth. Here we present a series of simulations that vary the atmospheric methane concentration and latitudes of west–east-oriented mountain ranges on an idealised supercontinent. For a given methane concentration, the latitudes of mountains control whether a Snowball Earth forms or not. Significantly, mountains in middle latitudes inhibited Snowball Earth formation, and mountains in low latitudes promoted Snowball Earth formation, with the supercontinent with mountains at ±30° being most conducive to forming a Snowball Earth because of reduced albedo at low latitudes. We propose that the extreme sensitivity of a Snowball Earth to reconstructions of the paleogeography and paleoatmospheric composition may explain the observed glaciations, demonstrating the importance of high-quality reconstructions to improved understanding of this early period in Earth’s history.

## Introduction

The Earth’s climate has three states: no ice caps (such as believed to have occurred during the warm Mesozoic), partial ice caps (as the present period of glacial and interglacials during the Pleistocene and Holocene), and an Earth completely covered in ice and snow called Snowball Earth. Earth is believed to have been in and out of the Snowball-Earth state during at least two periods, the NeoProterozoic Cryogenian Period (720–635 million years ago)^1,2^ and the PaleoProterozoic glaciation (2.45–2.2 billion years ago)^3–7^.

The PaleoProterozoic glaciation occurred during a period of climatic change on Earth. To compensate for the Sun being about 19% weaker than present during the PaleoProterozoic^8^, higher concentrations of greenhouse gases, such as carbon dioxide, methane, and water vapor, must have been present in the atmosphere to maintain a temperate climate^9–13^. For example, atmospheric methane concentrations were perhaps as much as 1000 times that of the present due to methanogenic archaea. Atmospheric oxygen concentration was increasing rapidly as a result of oxygen production by photosynthetic bacteria exceeding the sinks of oxygen^14,15^. With the increasing oxygenation of Earth, methane reacted with the oxygen, producing carbon dioxide and water vapor. These bacteria would have been a sink for the carbon dioxide, as well. In addition, weathering of fresh basaltic surfaces from rifting of the supercontinent would have reduced the amount of carbon dioxide in the atmosphere^16,17^. As carbon dioxide and water vapor are less powerful greenhouse gases compared to methane, this weakened the Earth’s greenhouse, possibly leading to Snowball Earth^14,18,19^.

Earth may have slipped in and out of glacial episodes three or four times during the PaleoProterozoic, culminating in the Makganyene Snowball Earth episode at about 2.3–2.2 billion years^4,18^. The evidence of cap carbonates at the end of some these glacial episodes suggests one reason for these cycles may be the emission of a large amount of carbon dioxide, perhaps from underwater volcanism, and an enhanced greenhouse effect that led to the end of Snowball Earth^7,20–22^. But, greenhouse gas concentrations are not the only control on Earth’s climate.

In addition to changes in the atmosphere during this period, the Earth’s continents came together in what was perhaps the first supercontinent, although paleogeographical reconstructions are necessarily uncertain given its age^23–27^. Collisions between various continents had produced mountain ranges^28,29^. Erosion of the mountains by precipitation would reduce atmospheric carbon dioxide, further weakening the greenhouse effect^16,17,30^. Thus, mountain formation due to continent–continent collisions during supercontinent assembly may also have led to fluctuations in the atmospheric greenhouse. Weathering affecting the atmospheric carbon dioxide concentration is not the only effect on the climate. The direct effect of the topography by changing the atmospheric circulation may have favored or inhibited the potential for the Earth to enter a snowball state. For example, the effect of the mountains at higher latitudes may have had a different impact on the Snowball Earth than mountains at lower latitudes. Such sensitivities have not been investigated previously for the PaleoProterozoic glaciation or any other Snowball Earth period. Given the uncertainties in the reconstruction of the continents and their mountains, exploration of these sensitivities may shed light on the conditions likely responsible for the glaciations.

**Figure 1:**
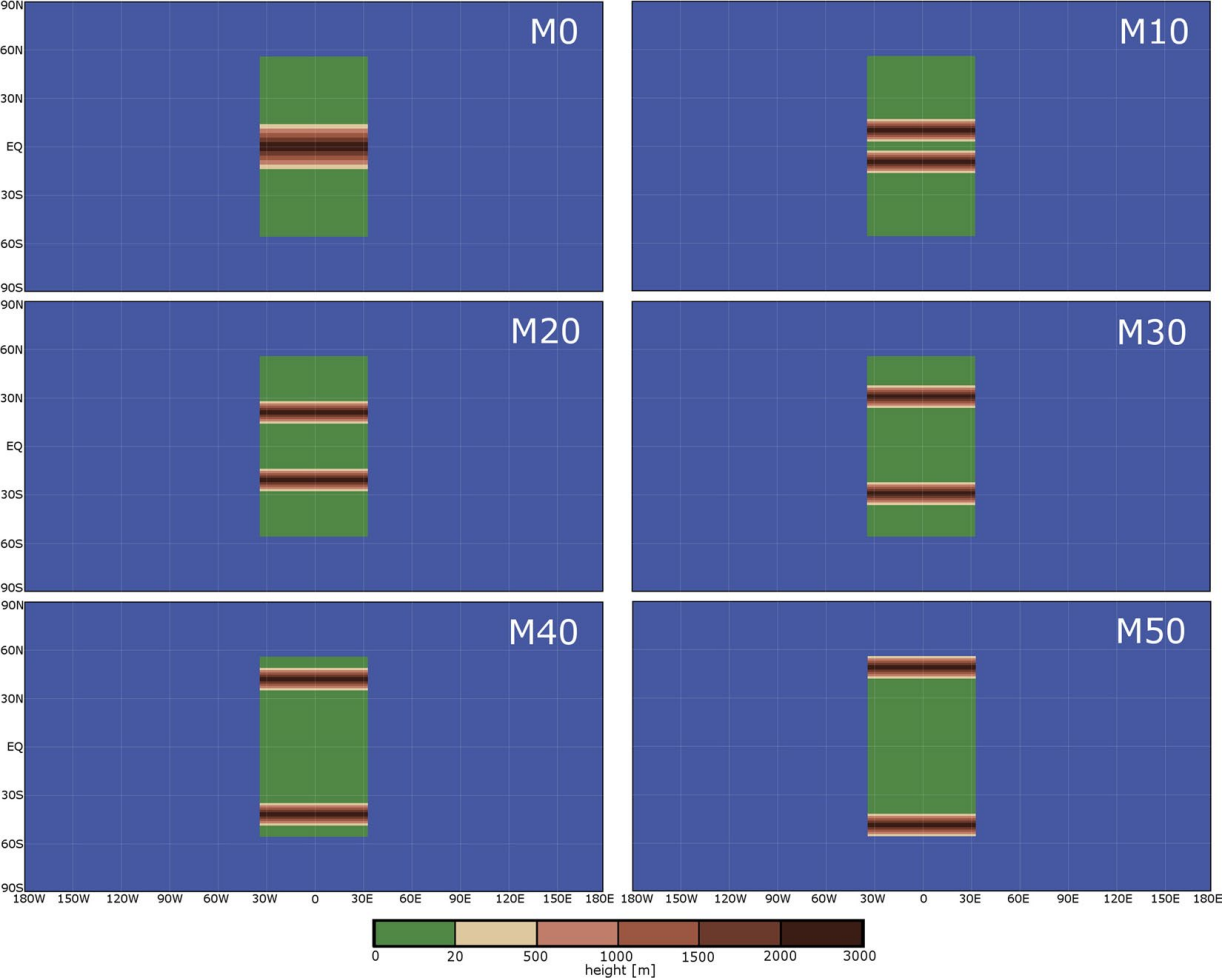
Terrain heights for simulations varying the latitudes of mountain ranges. Colors indicate the terrain elevation, with a maximum terrain height of 3 km.

Here we present a series of paleoclimate model simulations to examine some of the sensitivities that affect the onset of Snowball Earth. The model is a coupled atmosphere–ocean climate model^31^ that has simulated Snowball Earths previously^32^. We consider an idealized supercontinent centered on the equator (Fig. 1). We perform a series of 49 simulations varying the atmospheric greenhouse gas concentration and latitudes of west–east-oriented mountain ranges, keeping all other components of the model fixed. We choose to fix carbon dioxide at 10,000 ppm and vary methane concentrations from 1 to 100 ppmv to regulate the magnitude of the greenhouse effect on Earth. Two mountain ranges are centered at various latitudes: ±10° (M10), ±20° (M20), ±30° (M30), ±40° (M40), and ±50° (M50), with one simulation having one mountain range on the equator (M0) and one simulation having no mountains at all (FLAT) (Fig. 1). Mountain ranges are 3 km high, 1550 km wide, and extend from the west to east coasts of the continent. Although the design of these simulations were meant to idealise the supercontinent and not represent any particular reconstruction, at least one paleoclimate reconstruction^17^ suggests that there might have been an orogeny at the equator, which would have been similar to simulation M0. Each simulation ran for 400 years by which the latitude of sea-ice extent reached an equilibrium, either at a latitude greater than about ±30° or at the equator (i.e., Snowball Earth). As in previous modelling studies using this model, if sea ice forms within about 25° latitude of the equator, then the climate ultimately proceeds to a Snowball-Earth state, and intermediate latitudes of maximum sea-ice extent do not form^17,32^. In reality however, this latitude may be lower because of the omission of sea-ice dynamics in the model^33–35^.

## Sensitivity of Snowball Earth to Mountain Location

**Figure 2:**
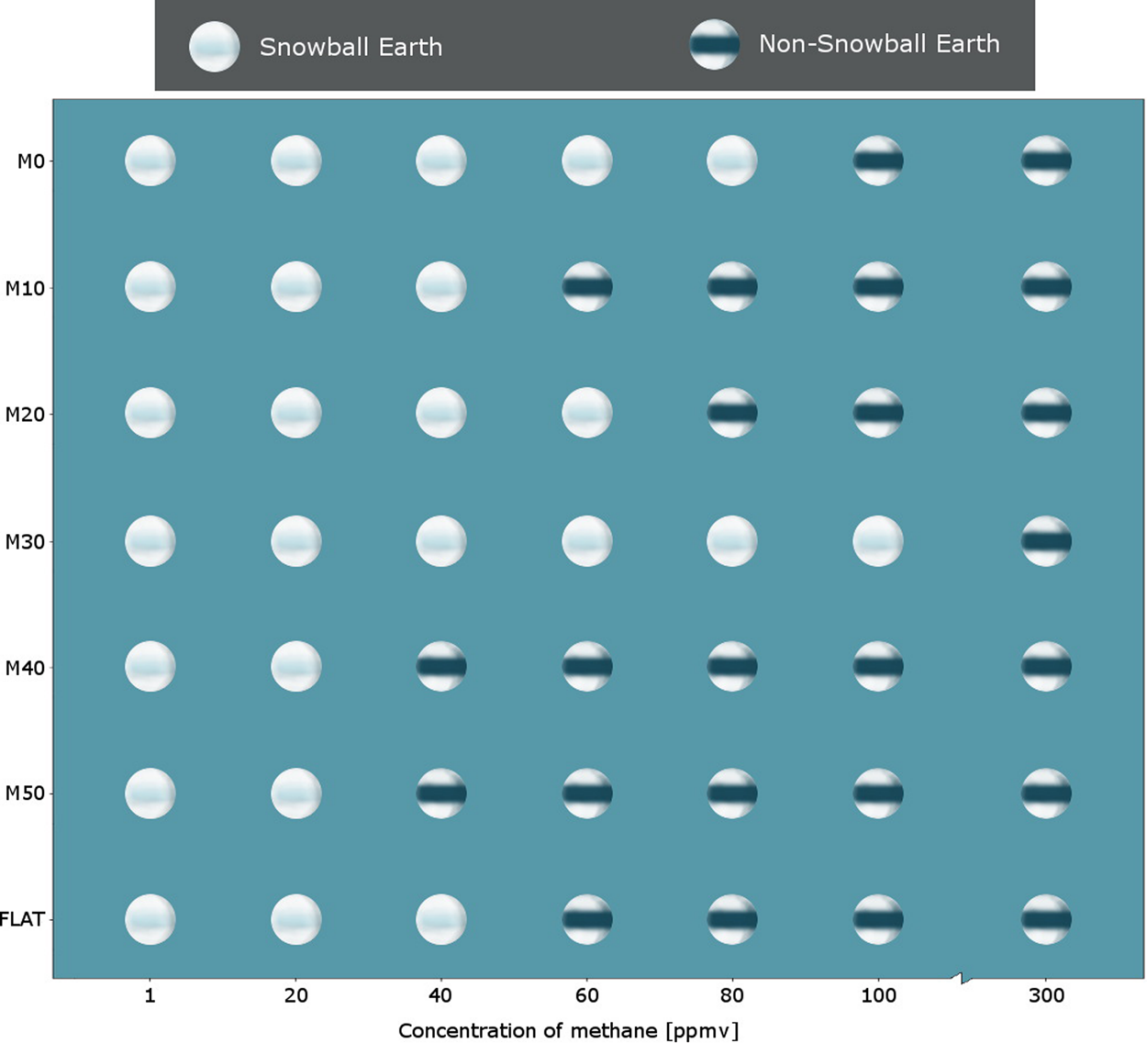
Summary of 49 simulations varying atmospheric methane concentration versus latitude of mountain ranges. White circles indicate simulations that form a Snowball Earth at equilibrium. Circles with dark equatorial band indicate simulations that develop ice caps at equilibrium.

In our model simulations, methane concentrations less than or equal to 20 ppmv produce Snowball Earths regardless of the configuration of the mountains, and methane concentrations 300 ppmv or more produce partially ice-covered Earths (Fig. 2). However, at intermediate methane concentrations of 40–100 ppmv, the latitudes of the mountain ranges affect whether a Snowball Earth forms or not. For example, mountains at ±40° and ±50° latitude and 40 ppmv of methane are sufficient to prevent a Snowball Earth compared to mountains at ±30° latitude where a Snowball Earth forms at 100 ppmv of methane. Mountain ranges in the tropics (0° to ±20°), as well as the flat supercontinent, form Snowball Earths at methane concentrations of 40–80 ppmv.

**Figure 3:**
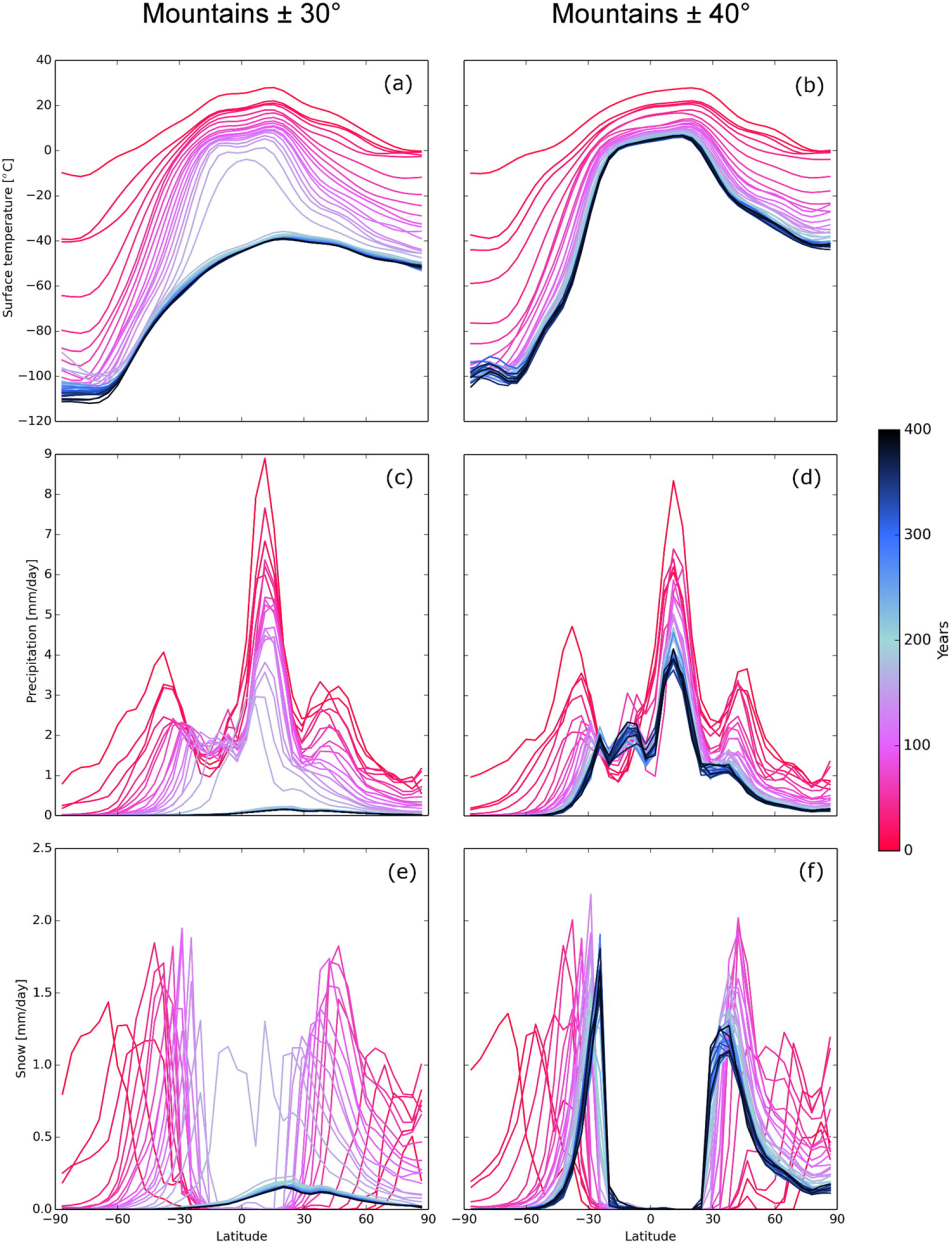
Evolution of the latitudinal profiles of July mean surface temperature, precipitation rate, and snowfall rate in M30 and M40 for methane concentrations of 40 ppm. Colored lines represent the July average every 10 years for simulations that form a Snowball Earth (M30) and that do not (M40).

Regardless of Snowball Earth formation or not, meridional profiles of zonally averaged monthly-mean surface temperature, precipitation rate, and snowfall rate as a function of latitude evolve similarly, up until the rapid transition to a Snowball Earth occurs (Fig. 3). In all cases, snow approaches the equator over time until equilibrium is reached (Fig. 3e,f). The maximum snowfall rate occurs at the boundary between the snow-covered and snowless latitudes, which also tends to be where the mean surface temperature is about 0 °C (Fig. 3a,b). The highest total precipitation rate is found at the Intertropical Convergence Zone (Fig. 3c,d), explaining why the maximum snowfall rate is at the closest latitude to the equator where snow can exist. For continents featuring mountain ranges, a smaller peak in snowfall rate appears at the latitude of the mountains. This increase in snowfall rate results in additional snow accumulating on the continent, increasing the albedo of Earth, and leading to cooling. Once in the Snowball Earth, precipitation and snowfall rates both drop due to the loss of surface moisture to feed precipitating systems (Fig. 3c,e).

When the mountains are at ±40° or ±50° latitude, the tropics remain above freezing, and cooling due to sea ice forming is limited to higher latitudes (Fig. 3b,d,f), explaining why these simulations are resistant to producing Snowball Earths except when methane is 20 ppmv or less (Fig. 2). Also, cold air from the polar regions is inhibited from reaching the tropics by the mountains at ±40° and ±50° latitude (Fig. 3b). As such, the tropics stay relatively warm compared to the other simulations, and Snowball Earths are inhibited from forming, even at relatively low methane concentrations.

When the mountains are at ±30° (Fig. 3a,c,e), snow falls on the mountaintops more equatorward than when the mountains are at ±40°. The snow-capped mountains reduce the temperature in the subtropics (Fig. 3a,e), making it easier for the subtropics to cool and for snow to form at higher methane concentrations. Thus, the temperature easily drops below zero and the Snowball Earth forms more easily (Fig. 3a,e). With mountains at even lower latitudes, greater cooling is more difficult because of the higher insolation preventing ice to form on the tropical mountaintops. Thus, Snowball Earths form at concentrations of 40–80 ppmv (Fig. 2).

These variations are explained by the snowfall on the mountains lowering the albedo. Sea ice forms when the atmospheric temperature is below freezing. Snow falling on the continent is more likely to remain snow if the temperature is below freezing. Once ice or snow is present, the albedo is reduced, which reduces solar insolation, starting the feedback process toward Snowball Earth. The impact of this additional snow on the radiation budget of the planet depends on its proximity to the equator (i.e. location of maximum insolation), and also to the latitude that snow extends to before a runaway ice-albedo feedback occurs.

Because temperature and snow/ice cover are intimately linked, we need a way to untangle that feedback. Thus, we perform a different set of simulations at 100 ppmv methane. At such high methane concentrations, only the simulation with mountains at ±30° produced a Snowball Earth (Fig. 2). In this new set of simulations, the mountain ranges were replaced by flat terrain with an albedo of snow instead of desert rock. The goal was to see if nonSnowball Earths could be changed to Snowball Earths by reducing the albedo in crucial latitudes instead of by raising the elevation of the terrain. Therefore, we test our hypothesis of whether the albedo changes led to the Snowball Earth or whether the changed dynamics of the atmospheric circulation due to the mountains was responsible. Our results show that reducing the albedo at ±40° or ±50° latitude does not affect the likelihood of Snowball Earth formation. Those latitudes are where the mountains are already snow-capped within 100 years into the simulations. In contrast, lowering the albedo at 0°, ±10°, or ±20° latitude from the start of the simulation results in formation of a Snowball Earth. This is because the albedo reduces insolation, cooling the tropics below freezing more easily and leading to further snowfall and albedo reduction. Thus, the increase in albedo in the tropics is crucial to the formation of Snowball Earth, not the location of the mountains affecting the planetary-scale circulation or how the precipitation distribution changes. That Snowball Earths can form at such high methane concentrations merely by changing the albedo indicates the sensitivity of the Earth’s climate at this time in geologic history.

## Model for Snowball Earth Formation in the PaleoProterozoic

The multiple phases of glaciation that occurred in the PaleoProterozoic have been attributed to changes in the atmospheric composition^5,6,17^. Our results show that even in the absence of changes in atmospheric composition, changes in the latitudes of mountains (or more precisely, the albedo) is sufficient to change the Earth from a nonSnowball state to a Snowball state. We propose that one or both of two mechanisms may have explained the glaciations observed in the geological record during the PaleoProterozoic. Continental drift of the supercontinent may move mountain ranges from one climate zone to another^24^, leading to a change in climate state, or new orogenies by continent–continent collisions may produce new mountains at latitudes leading to a change in Earth’s climate state. Specifically, raising a mountain in lower latitudes in an unglaciated state may cause ice to form more easily, leading to a glaciation. What is surprising is that such an extreme climate of an Earth covered in ice could occur by relatively small and subtle changes in atmospheric composition or paleotopography and paleogeography, especially for a supercontinental landmass that occupies only 15% of the Earth’s surface and extends across 68° of longitude.

While these results show extreme sensitivity to the position of mountains and Snowball Earth formation, other results on a modern Earth show that the modern topography stabilizes the Earth against forming a Snowball-Earth state so easily^36^. Changes in the topography can also lead to changes in the ocean circulation, reversing the thermohaline circulation^37^. These results also may pertain to exoplanets where the flatter surfaces of exoplanets larger than Earth may stabilize the exoplanet against forming an ice-covered state.

In summary, the PaleoProterozoic episodes of glaciation led to important geological and biological changes on Earth. Evidence from numerical model simulations indicates the strong sensitivity to the paleogeography and that differences of tens of ppmv of methane or of 10° latitude in the mountains can lead to the difference between a Snowball Earth or not. We propose that the multiple episodes of glaciation during the PaleoProterozoic were controlled by fluctuations in the paleotopography, paleoalbedo, carbon dioxide, and methane concentrations. As such, our results constitute testable hypotheses and further constraints on the paleoenvironments that were possible during the PaleoProterozoic.

## Methods

### The FOAM model

The climate model Fast Ocean Atmosphere Model (FOAM) comprises an atmosphere and ocean component that are coupled to each other^31^. Although simplified to run a large number of simulated years per day, it is a complex climate model that solves time-dependent equations describing the fluid and thermodynamics of the system. FOAM is a coupled general circulation model, meaning that it uses different circulation models for various aspects of the simulation. The Community Climate Model CCM3 is used to simulate the atmospheric components of the planet, whereas the Modular Ocean Model OM3 is used to simulate the dynamics of the ocean. The two components are coupled, meaning that the transfer of heat and moisture at the boundary is calculated, as well as the distribution of any sea ice. Although the dynamics of the sea ice is not included in FOAM, other studies have examined how the inclusion of sea-ice dynamics changes the ability of a model to initiate Snowball Earth^33–35^. In particular, when polar ice caps become large, they flow under the influence of gravity, facilitating the advance of the ice front equatorward. This result means that Snowball-Earth conditions would onset at a lower value of methane concentration for models with sea-ice dynamics than for those without. In our simulations, which are heavily abstracted, this effect does not change the qualitative conclusion that the topography of the Earth (or more specifically, the albedo) affects the onset of Snowball Earth.

FOAM splits the atmosphere into 18 vertical levels, each with a depth in units of pressure of 55 hPa. The atmosphere has 40 latitudinal and 48 longitudinal grid points. The ocean model has a higher resolution, with 128 latitudinal and longitudinal grid points with a maximum of 20 vertical levels depending on the chosen ocean depth. These levels are not equally spaced, with the first 14 levels making up the top 1500 m of the ocean. For simplicity, the ocean depth is set to a constant value of 3100 m, one of the fixed depths available in the ocean model. This depth is similar to the average ocean depth on present-day Earth. The flat continent had a constant elevation of 20 m above sea level. The area of the oceans and continents does not change when water from the oceans is removed to form ice sheets on land. The depth of the continental ice sheets does not affect the continental topography.

### Astronomical parameters and atmospheric composition

For simplicity, our simulations share the same orbital characteristics (i.e., orbital rotation rate, eccentricity, obliquity, date of perihelion) as the present day, with the exception of a 360-day year. The solar constant is set to the predicted solar luminosity 2.4 billion years ago of 1100 W m^−2^, 19% lower than the present-day value^8^.

To compensate for this lower solar constant, the concentration of greenhouse gases in the atmosphere was higher than the present-day values^10^. Greenhouse gases including carbon dioxide and methane have been hypothesised to have created a strong greenhouse effect on early Earth. This experiment assumes that carbon dioxide is the largest contributor to this warming. One possible range of carbon dioxide concentrations during the Proterozoic is 4000 to 80,000 ppmv^38^. A value of 10,000 ppmv is chosen, a number within this range and also used in another study^17^. The range of atmospheric methane concentration to test over was decided based on estimates^10^ and the results of modelling^17^. This range is 1–100 ppmv.

### Continental configuration

The size and location of the continents 2.4 billion years ago is uncertain. One plausible reconstruction places a supercontinent close to the equator, with a larger meridional dimension than zonal one^25^. Another constructed simulation from the PaleoProterozoic glaciation used a similar series of continents^17^. A box containing the entire supercontinent occupies about 15% of Earth’s total surface area. To replicate this, a rectangular continent (on a latitude–longitude map projection) centred on the equator that is 15.6% of the total surface area is used in this experiment. This continent extends from 56°N to 56°S, and has a west–east width of 68° longitude. The continent surface type is set to desert rock, which has an albedo of 24%^39^, replicating the plant-less continent surface of 2.4 billion years ago. The elevation of the continent is 20 m above sea level.

### Mountain configuration

Little information is available about the topography of this supercontinent, and so we experiment with a number of hypothetical sets of mountain ranges at various latitudes (Fig. 1). They are all placed parallel to latitude lines, and the pairs are placed the same distance from the equator in each hemisphere. The mountains are approximately Gaussian in profile, have a latitudinal width of 10 grid points, which is equivalent to about 1550 m (14° latitude) at the equator, and each mountain spans the entire longitudinal dimension of the continent. The mountain dimensions are restricted by the 128 × 128 grid boxes, which limits how gradual the slope of the mountains can be, but they have a maximum height of 3000 m. A suite of model simulations varying the maximum height of the mountains using the M40 and M50 configurations showed no sensitivity to the onset of the Snowball Earth for mountain heights between 0.5 and 3.0 km. Such results are consistent with those where we vary the albedo of flat terrain at low latitudes and show that the effect of the albedo on climate is greater than the effect of the terrain on climate.

### Initialisation and simulation

The simulations are performed in a time-slice experiment where the model is initialised and then run until it reaches equilibrium. Each simulation is initialised with the conditions of the present climate. The polar surface temperature is set to 10 °C and the equatorial temperature to 28 °C. The conditions being tested cannot maintain such a high surface temperature, and so the system evolves to a new, colder state. The state that the system ends up in primarily depends on the tested value of methane. Simulations are run for a total of 400 years to give the climate enough time to evolve to its new state. But, this time is not sufficient to produce ice sheets of substantial depth to rival the height of the mountain range or reach the stage where the ice would flow a substantial distance due to gravity (which could not be modeled in FOAM anyway). Thirty-day mean values are output from the model 12 times each model year. These data describe the monthly-mean state of the atmosphere, ocean, and cryosphere (ice and snow) for that year.

### Sensitivity to initial conditions

In addition to the 49 simulations performed in the study, 40 other simulations were performed with a continent comprising 27% of the area of the Earth (comparable to modern values), solar constant of 1142 W m^−2^ and a carbon dioxide concentration of 2000 ppm. Methane was varied from 100, 200, 400, 600, 800, 1000, 2400, and 2600 ppm. These simulations show a similar sensitivity to that presented in the present article.

## Data Availability

The datasets generated and analysed during the current study are available from the corresponding author on reasonable request.
